# Purpura vasculaire révélant une granulomatose avec polyangéite: à propos d'un cas

**DOI:** 10.11604/pamj.2015.21.111.7120

**Published:** 2015-06-10

**Authors:** Olfa Berriche, Sonia Hammami

**Affiliations:** 1 Service de Médecine Interne, Hôpital Mahdia, Tunisie; 2 Service de Médecine Interne, Hôpital Monastir, Tunisie

**Keywords:** Purpura vasculaire, granulomatose, polyangéite, purpura, granulomatosis, polyangiitis

## Image en medicine

La granulomatose avec polyangéite (GPA) est une vascularite systémique rare, caractérisée par une atteinte des voies aériennes supérieures, des poumons et des reins; cependant de nombreux organes peuvent être touchés. Les lésions cutanées sont peufréquentes, rarement inaugurales et de mécanismes mal élucidés. Nous rapportons une observation originale par la survenue d'un purpura vasculaire s'intégrant dans le tableau clinique inaugural d'une granulomatose avec polyangeite. Il s'agit d'une patiente âgée de 52 ans, admise pour bilan étiologique d'un purpura vasculaire nécrotique, infiltré prédominant au niveau des deux jambes. A l'interrogatoire: notion de sinusites à répétition depuis une année, des polyarthralgies des grosses articulations de type inflammatoire et des épigastralgies. La biopsie cutanée a objectivé une vascularite leucocytoclasique, avec immunofluorescence directe négative. La recherche des ANCA était positive de type c-ANCA. L'examen ORL objectivait des ulcérations nasales. Le bilan biologique objectivait un syndrome inflammatoire biologique, le bilan rénal montrait une hématurie microscopique, une protéinurie à 0,7 g/24h et une créatinine à 205 µmol/l. La biopsie rénale trouvait une glomérulonéphriteextra-capillaire à dépôt exsudatif de PNN. La radiographie thoracique montrait des infiltrats diffus réticulo nodulaires. Le diagnostic de GPAétait ainsi retenudevant la présence de 3 critères de l'ACR: inflammation nasale, hématurie et anomalie de la radiographie pulmonaire. Un traitement par corticothérapie associée à des bolus bimensuels de cyclophosphamide en intraveineux à la dose de 14 mg/kg, soit 12 cures était instauré. L’évolution était marquée par la disparition du purpura au bout de trois semaines, et l'amélioration de la fonction rénale.

**Figure 1:**
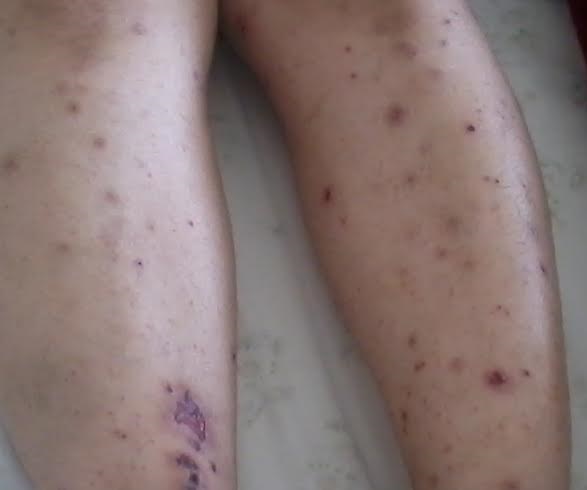
Purpura vasculaire infiltré et nécrotique étendu au niveau des deux jambes

